# High anti-Müllerian hormone levels might not reflect the likelihood of clinical pregnancy rate in IVF/ICSI treatment

**DOI:** 10.5935/1518-0557.20200094

**Published:** 2021

**Authors:** Tie-Cheng Sun, Shan-Jie Zhou, Ling-Li Song, Jian-Hua Li, Xi Chen, Li Tian

**Affiliations:** 1 Reproductive Medical Center, Department of Obstetrics and Gynecology, Peking University International Hospital, Beijing, 102206, China; 2 Reproductive Medicine Centre Peking University Second Affiliated Hospital, Beijing, 100044, China; 3 Center of Reproductive Medicine and Genetics, Seventh Medical Center of PLA General Hospital, Beijing 100027, China

**Keywords:** anti-Müllerian hormone (AMH), number of retrieved oocytes, good quality embryos (GQEs), clinical pregnancy rate (CPR), IVF/ICSI

## Abstract

**Objective:**

To investigate if high anti-Müllerian hormone (AMH) concentration is a useful tool to predict the outcome of assisted reproductive treatment.

**Methods:**

Retrospective cohort study involving 520 patients who underwent IVF/ICSI procedures in a university hospital. We measured the serum AMH level on day 3 of the menstrual cycle. Based on AMH levels, we divided the patients into three groups as follows: low (<25th percentile) AMH group, average (25th to 75th percentile) AMH group and high (>75th percentile) AMH group. We recorded the fertilization rate (FR), the number of oocytes retrieved, the number of good quality embryos (GQEs) and the clinical pregnancy rate (CPR).

**Results:**

There was no difference between the three AMH groups in terms of maternal age, body mass index (BMI), follicle-stimulating hormone (FSH), estradiol (E2), luteinizing hormone (LH) and testosterone (T) in the IVF/ICSI cycles. The women in the high serum AMH group had a higher number of retrieved oocytes than those in the low or average AMH groups (*p* < 0.01) in the IVF/ICSI cycles. Compared with the low or average AMH groups, the women with high AMH levels had a higher number of good quality embryos (GQEs) in the IVF/ICSI cycles (*p* < 0.01). However, high AMH women had no significantly higher clinical pregnancy rate (CPR) compared to the women in the low or average AMH groups. In addition, for the prediction of CPR, the AMH levels alone were not an independent predictor of CPR for IVF and ICSI cycles in the ROC curve analysis.

**Conclusions:**

High anti-Müllerian hormone levels are an independent predictor of the number of retrieved oocytes and good quality embryos (GQEs), but might not reflect the likelihood of higher clinical pregnancy rates (CPR) in IVF/ICSI treatment.

## INTRODUCTION

IVF cycle outcomes are associated with ovarian reserve, number of retrieved oocytes and good quality embryos (GQEs) (Sunkara *et al*., 2014). Widely-used markers of ovarian reserve, such as baseline follicle-stimulating hormone (FSH), estradiol (E2), inhibin B, antral follicle count (AFC) and ovarian volume, were effective in IVF/ICSI treatment (Erdem *et al*., 2004; Shahrokh Tehraninezhad *et al*., 2016). Usually, the baseline day 3 serum FSH level is used to predict ovarian reserve, and levels >10 IU/L are consistent with poor ovarian response (Pankhurst, 2017). However, baseline serum FSH is not the best predictor of ovarian response, number of retrieved oocytes and good quality embryos (GQEs) (Nelson 2013; Vural *et al*., 2014; Pankhurst 2017).

The anti-Müllerian hormone (AMH) has been identified as one of the most widely used promising biochemical makers for the detection of ovarian response, and it has been extensively used for *in vitro* fertilization cycles (Gomez *et al*., 2016). Several cross-sectional studies suggest an association between different AMH cut-off values and IVF/ICSI outcomes (Kotanidis *et al*., 2013; Lehmann *et al*., 2014). In the literature on AMH levels, the relative importance of pregnancy rate has been subjected to considerable discussion (Goswami & Nikolaou, 2017). In recent years, researchers have shown an increased interest in embryo quality. There is increasing concern that AMH levels are advantageous for oocyte and embryo quality (Lehmann *et al*., 2014; Gleicher *et al.*, 2016a; 2016b; Bhide *et al*., 2017). However, some studies have indicated that AMH levels predict ovarian responsiveness, but not embryo quality or clinical pregnancy in IVF/ICSI cycles (Smeenk *et al*., 2007; Lamazou *et al*., 2011). These studies, though compelling, are limited by different AMH cut-off values and these differences limit their external validity. Although extensive research has been carried out on AMH levels and ART outcomes, the question whether AMH level as a biochemical marker is a better predictor of GQEs remains highly controversial, leaving clinical specialists with limited evidence to guide ovarian stimulation. There is patient data from different countries, and their AMH cut-off values might have individual/ethnic differences (Nelson *et al*., 2020). Therefore, dividing patients into groups based on low, average and high AMH levels is more scientific. To date, few studies have investigated the association between (low, average and high) AMH levels and good quality embryos (GQEs).

Therefore, the goal of our study was to investigate whether high anti-Müllerian hormone (AMH) concentration is a useful tool to predict the outcome of assisted reproductive treatment.

## MATERIALS AND METHODS

### Patients

About 520 patients who received IVF (n = 302) / ICSI (n = 218) procedures between September 2015 and February 2017 at Peking University People’s Hospital were recruited. The Medical Ethics Committee of the Peking University People’s Hospital (No. 2015- 87) approved the procedure.

The criteria for selecting the subjects were as follows: all women who underwent IVF/ICSI cycles with GnRH agonist treatment, aged <43 years had normal size and shape of uterus and ovaries, as per examined using the ultrasound. The exclusion criteria included being an oocyte donor; women with suspicious ovarian malignancies; no embryo transfer; endocrine disorders; genetic or reproductive system diseases, including polycystic ovary syndrome (PCOS).

Each patient signed a written consent for before treatment and had normal gynecological ultrasound (uterus, ovary and pelvis). The infertility etiology included a variety of causes, such as tubal factors, endometriosis, idiopathic causes and male factors. The semen parameters were evaluated according to WHO guidelines for the time of liquefaction, volume, pH, concentration, progressive motility, non-progressive motility and morphology (5th edition)(Ford, 2010).

The women were divided into three groups according to the percentile of serum AMH levels: <25^th^, 25^th^ to 75^th^, and >75^th^. Data on AFC, number of oocytes collected and number of available embryos were included in this study.

### ART procedure and pregnancy assessment

All patients received ovarian stimulation using a standard protocol as we described (Corfman *et al*., 1993; Palmer *et al*., 2011; Sun *et al*., 2018). In brief, the protocol began with daily subcutaneous injections of Triptorelin Acetate (3.75 mg/14-21d; Ipsen Pharma, France) during their prestimulation cycle. Follicular development was stimulated using recombinant human follicle stimulating hormone (hFSH, Merck Serono, Germany). Subsequently, the dose of hFSH was adjusted according to the ovarian response. If the maximum diameter of two or more follicles reached 17 mm, we administered human chorionic gonadotropin (250µg; hCG, Merck Serono, Germany). The oocyte retrieval was collected through the vagina 34-36 hours after hCG injection. Oocyte retrieval was performed 36-38 hours after human chorionic gonadotropin (hCG) administration and embryo scoring. Embryos were based on previous descriptions (Sun *et al*., 2018). Embryo morphologywas evaluated on days 3, 5-6 at standard time points according to the Istanbul consensus (Alpha Scientists in Reproductive Medicine & ESHRE Special Interest Group of Embryology, 2011). Good quality embryos on day 3 after egg collection are defined as having 6-8 cells, <10% fragmentation or above using the agreed grading system (Alpha Scientists in Reproductive Medicine & ESHRE Special Interest Group of Embryology, 2011). Good embryo blastocyst quality (Gardner grade ≥3BB) was assessed based on expansion, trophectoderm (TE), and inner cell mass (ICM) (Jacobs *et al*., 2020). One or two embryos were transferred on day 3/5 of oocyte retrieval. The luteal phase was supported with a daily 60 mg intramuscular injection of progesterone, prior to embryo transfer (42-72 hours after oocyte pick-up).

On the 14th day after embryo transfer, serum β-hCG levels of >5 IU/L were defined as a positive outcome (biochemical pregnancy). Clinical pregnancy was defined as the presence of a visible fetal heartbeat under transvaginal ultrasonography, 4 weeks after embryo transfer.

### Transvaginal sonography (TVS) measurements

One physician, using a PHILIPS HD11XE ultrasound system to determine the follicle’s diameter, performed all ultrasound examinations. All antral follicles between 2 and 8 mm in diameter were measured and counted. The total number of follicles in both ovaries was defined as the total antral follicle count (AFC). The antral follicle count (AFC) was assessed through transvaginal ultrasound on days 2-4 of the menstrual cycle.

### AMH assay

Centralized serum AMH levels were measured using an AMH detection kit, in accordance with the manufacturer’s instructions (Elecsys^®^ from Roche AMH assay. Roche Diagnostics, Mannheim, Germany) on day 3. The coefficients of variability (CV) for AMH level were functional sensitivity, 0.2 ng/mL; intra-assay CV, 4%; and inter-assay CV, 8%. All values are expressed in ng/mL.

### Statistical Analysis

Data management and analysis were performed using the SPSS (version 18.0) for Windows (SPSS Inc., Chicago, USA). The mean differences among the groups were analyzed using the independent samples t-test. The differences between variables were measured using the Kruskal-Wallis test. The ROC curves for variables were created according to ROC analyses. The *p*-value descriptive data was generated for all variables. All p values were two-sided, and a *p* value of *<*0.05 was considered statistically significant.

## RESULTS

### Patient characteristics

We had 520 individuals included in this study. AMH levels ranged from 0.1 to 10.00 ng/ml, with a mean (SD) of 3.30±2.553 ng/ml. The results were obtained based on the characteristics of the patients undergoing IVF/ICSI treatment; with low, average and high AMH levels, and they are summarized in Table 1 and Table 2. For IVF cycles, the mean age of the infertile women included in this study was 38.0±3.78, 38.57±4.14 and 37.07±4.54 years, respectively, for low, average and high AMH level groups on day 3. Other characteristics in IVF cycles were as follows: AFC: 8.75±4.12, 12.2±6.77 and 14.09±7.85; Number of retrieved oocytes: 7.67±4.76, 10.42±4.65 and 14.99±7.34; GQEs: 3.33±2.1, 3.99±2.75 and 5.08±3.35.

**Table 1:** Characteristics of patients (undergoing IVF treatment) with low (<25th percentile), average (25th to 75th percentile) and high (>75th percentile) levels of serum AMH on day 3

Characteristics	AMH (ng/ml)		
	<1.495	1.495-4.975	>4.975
N	75	152	75
Age (y)	38.0±3.78	38.57±4.14	37.07±4.54
Duration of infertility (y)	5.38±4.33	4.64±3.51	4.25±3.12
**AFC**	**8.75±4.12**	**12.2±6.77**	**14.09±7.85ab**
BMI (kg/m^2^)	23.91±5.62	23.04±3.55	23.42±4.28
E2 (pg/ml)	130.42±262.89	140.69±413.69	117.17±148.61
FSH (ng/ml)	8.24±5.03	7.37±2.84	7.68±5.03
LH (ng/ml)	4.11±2.82	4.04±2.24	7.1±1.11
T (ng/ml)	0.41±0.26	1.83±1.11	0.43±0.19
**Number of retrieved oocytes (n)**	**7.67±4.76**	**10.42±4.65**	**14.99±7.34^a^^b^^,^^a^^c^**
FR (%)	81.48±16.19	78.65±17.33	80.0±16.45
GQEs (n)	**3.33±2.1**	**3.99±2.75**	**5.08±3.35^a^^b^^,^^a^^c^**
CPR (%)	44.74(17/38)	50.72(35/69)	57.69(15/26)

Data expressed as mean ± standard deviation. The Kruskal-Wallis test was used and a *p* value <0.05 was considered statistically significant.

AFC: antral follicle count; BMI: body mass index; E2: estradiol; FSH: follicle-stimulating hormone; LH: luteinizing hormone; T: testosterone; FR: fertilization rate; GQE: number of good quality embryos; CPR: clinical pregnancy rate.

a Kruskal-Wallis multiple comparison test was used to determine which group differed from others.

b <.001, low versus high.

c <.001, average versus high.

**Table 2:** Characteristics of patients (undergoing ICSI treatment) with low (<25th percentile), average (25th to 75th percentile) and high (>75th percentile) levels of serum AMH on day 3

Characteristics	AMH (ng/ml)		
	<1.17	1.17-4.33	>4.33
N	55	110	53
Age (y)	36.07±3.37	36.81±4.4	36.38±4.65
Duration of infertility (y)	5.65±3.95	5.42±4.69	4.94±3.63
**AFC**	**11.91±5.11**	**13.17±6.33**	**18.75±6.54^a^^b^^,^^a^^c^**
BMI (kg/m^2^)	23.35±4.81	23.25±3.93	24.01±2.83
E2 (pg/ml)	70.99±65.19	98.33±196.34	168.03±692.18
FSH (ng/ml)	6.71±2.74	6.55±4.26	5.4±2.23
LH (ng/ml)	4.32±2.54	4.47±3.92	3.26±2.35
T (ng/ml)	0.34±0.22	1.14±3.75	0.57±0.26
**Number of retrieved oocytes (n)**	**7.09±3.04**	**13.17±6.33**	**18.53±8.65^a^^b^^,^^a^^c^**
FR (%)	73.87±21.01	73.03±18.08	66.59±16.37
GQEs (n)	**2.72±1.6**	**4.03±3.46**	**4.9±4.31^a^^b^**
CPR (%)	32(8/25)	34.55(19/55)	42.31(11/26)

Data expressed as mean ± standard deviation. Kruskal-Wallis test was used and a *p* value <0.05 was considered statistically significant.

AFC: antral follicle count; BMI: body mass index; E2: estradiol; FSH: follicle-stimulating hormone; LH: luteinizing hormone; T: testosterone; FR: fertilization rate; GQE: number of good quality embryos; CPR: clinical pregnancy rate.

a Kruskal-Wallis multiple comparison test was used to determine which group differed from others.

b <.001, low versus high.

c <.001, average versus high.

For ICSI cycles, the mean age of the infertile women in this study was 36.07, 36.81 and 36.38 years, respectively, for the low, average and high AMH level groups on day 3(Table 2). Other characteristics of the ICSI cycles were as follows: AFC: 11.91±5.11, 13.17±6.33 and 18.75±6.54; Number of retrieved oocytes: 7.09±3.04, 13.17±6.33 and 18.53±8.65; GQEs: 2.72±1.6, 4.03±3.46 and 4.9±4.31.

### Comparisons of fertilization rate, number of retrieved oocytes and GQEs

**Figure 1:**
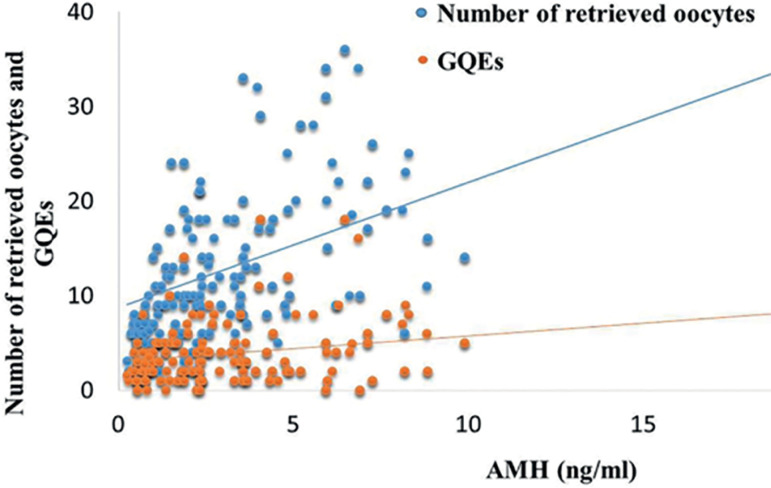
The correlation between NOC, GQEs and AMH levels

As expected, there were significant positive correlations between serum AMH levels and AFC (Table 1 and Table 2). Along with successive increases in AMH concentrations, the number of retrieved oocytes and the number of GQEs further increased. Figure 1 shows the correlation between the number of retrieved oocytes, GQEs and AMH levels. However, there was no significant correlation between AMH and FR in IVF or ICSI cycles.

Interestingly, the IVF results were associated with the ICSI cycles. Patients with higher serum AMH levels on day 3 also had higher numbers of GQEs (4.9±4.31 vs 2.72±1.6 and 4.03±3.46) than patients with low or average AMH levels (Table 2). In the ICSI group, although GQEs was statistically different among the three groups, CPR and AMH levels had no statistical difference in each group. In addition, there was no statistically significant difference based on age, body mass index (BMI), E2, LH, FSH or T levels, at different AMH levels.

### ROC curve analysis of AMH and CPR

**Figure 2:**
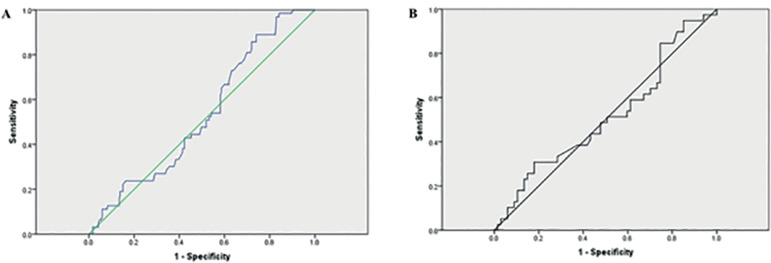
ROC curve analysis of AMH and CPR in IVF/ICSI cycles

In the ROC curve analysis, we tested the AMH for its ability to predict CPR in IVF/ICSI cycles. Figure 2A shoes, using the cut-off value of 1.365ng/ml, sensitivity of 90.5%, specificity of 37.7, and the ROC_AUC_ of 0.532 for the IVF cycles; while for ICSI cycles, using the cut-off value of 4.415 ng/ml, had a sensitivity of 30.8%, specificity of 82.2, and the ROC_AUC_ was 0.515 (Figure 2B).

## DISCUSSION

As mentioned in the literature review, various parameters (age, AFC, FSH, LH, estradiol and Inhibin B levels, and ovarian volume) have been used to assess ovarian response (OR). Even though the FSH level has been the OR biomarker, FSH, especially single day 3 FSH level measurements, may not be the best option for an accurate marker (Bukman & Heineman 2001; Erdem *et al*., 2004). The antral follicle count (AFC) exhibit sufficient inter-cycle reliability and inter-observer reliability for measuring ovarian reserve. However, AFC can be overestimated owing to the inclusion of atretic follicles; therefore, it does not have a prognostic value for the number of retrieved oocytes and the number of available embryos (*Mayo et al*., 2005). Other makers including LH, estradiol and Inhibin B levels are weaker than the FSH level and the AFC (Erdem *et al*., 2004). Previous studies have focused on AMH as a marker of OR for ovarian stimulation and ART outcome (Broekmans *et al*., 2006). However, the predictive value of AMH for the number of retrieved oocytes, FR, blastocyst formation rate, embryo quality, CPR and LBR remain controversial.

Some previous investigations have demonstrated the ability of AMH levels to predict fertilization rates and embryo quality for IVF/ICSI cycles (Hazout *et al*., 2004; Broer *et al*., 2013; Nelson, 2013). Therefore, AMH might be a good marker for the number of retrieved oocytes and embryo quality following IVF/ICSI. However, other studies have indicated that AMH levels are unable to predict number of retrieved oocytes and embryo quality (Smeenk *et al*., 2007). The results of our study suggest that AMH levels do not strongly correlate with AFC or number of retrieved oocytes for IVF or ICSI cycles, which confirms results of previous studies (Smeenk *et al*., 2007). However, a larger number of oocytes were collected when patients had average or high AMH levels; FR and number of retrieved oocytes increases along with an increase in AMH concentration.

Traditionally, some reports have shown that AMH levels may be used to reflect good quality embryos (GQEs), while AMH and AFC have been shown to be predictors of the number of retrieved oocytes and number of GQEs available for transfer and freezing (Majumder *et al*., 2010). Different serum AMH concentrations have also been associated with oocyte quality, embryo development parameters and IVF/ICSI outcomes (Irez *et al*., 2011). Other studied have shown that AMH levels have no direct effect on embryo quality (Smeenk *et al*., 2007). In the present study, higher levels of AMH are correlated with number of GQEs for IVF/ ICSI cycles. These results match those reported in earlier studies (La Marca *et al*., 2010; Scheffer *et al*., 2018). Patients with high serum AMH levels in our study had a higher CPR in IVF/ICSI cycles, when compared with those with low and average levels. These results further support the idea that AMH levels reflect the number of GQEs. In our study, we found that high AMH levels had no correlation with the number of CPR in IVF/ICSI cycles. This might be because AMH levels indicate the quantity of follicles, but it does not rule out the likelihood of “compromised quality”. Therefore, patients with higher AMH levels may still fail to conceive, even when more GQEs have been used. Most IVF and ICSI programs rely on the number of GQEs to increase success rates. However, more GQEs do not necessarily lead to better ART outcomes. Therefore, one of the issues that emerges from these findings is that AMH levels are not an independent maker that can be used to predict the number of CPR for IVF/ICSI cycles.

Our findings indicate that high AMH levels are not only becoming the most reliable biomarker in predicting ovarian response, but it may also help predict the number of good quality embryos (GQEs) in women who have undergone controlled ovarian stimulation for IVF/ICSI treatment. However, the relationship between high AMH levels and CPR for IVF/ICSI cycles need to be further confirmed through larger clinical studies.

## CONCLUSION

Our study shows that high anti-Müllerian hormone concentrations alone can be an independent predictor of the number of retrieved oocytes and good quality embryos (GQEs) in IVF/ICSI cycles. However, high anti-Müllerian hormone levels might not reflect the chance of clinical pregnancy in IVF/ICSI treatment.

## References

[r1] Alpha Scientists in Reproductive Medicine and ESHRE Special Interest Group of Embryology (2011). The Istanbul consensus workshop on embryo assessment: proceedings of an expert meeting. Hum Reprod.

[r2] Bhide P, Escriba M, Srikantharajah A, Joshi H, Gudi A, Shah A, Acharya G, Homburg R (2017). Anti-Mullerian hormone (AMH) and embryo quality assessed by time-lapse imaging (TLI): a cross-sectional observational study. Arch Gynecol Obstet.

[r3] Broekmans FJ, Kwee J, Hendriks DJ, Mol BW, Lambalk CB (2006). A systematic review of tests predicting ovarian reserve and IVF outcome. Hum Reprod Update.

[r4] Broer SL, Dólleman M, Van Disseldorp J, Broeze KA, Opmeer BC, Bossuyt PM, Eijkemans MJ, Mol BW, Broekmans FJ, IPD-EXPORT Study Group (2013). Prediction of an excessive response in in vitro fertilization from patient characteristics and ovarian reserve tests and comparison in subgroups: an individual patient data meta-analysis. Fertil Steril.

[r5] Bukman A, Heineman MJ (2001). Ovarian reserve testing and the use of prognostic models in patients with subfertility. Hum Reprod Update.

[r6] Corfman RS, Milad MP, Bellavance TL, Ory SJ, Erickson LD, Ball GD (1993). A novel ovarian stimulation protocol for use with the assisted reproductive technologies. Fertil Steril.

[r7] Erdem M, Erdem A, Gursoy R, Biberoglu K (2004). Comparison of basal and clomiphene citrate induced FSH and inhibin B, ovarian volume and antral follicle counts as ovarian reserve tests and predictors of poor ovarian response in IVF. J Assist Reprod Genet.

[r8] Ford WC (2010). Comments on the release of the 5th edition of the WHO Laboratory Manual for the Examination and Processing of Human Semen. Asian J Androl.

[r9] Gleicher N, Darmon SK, Kushnir VA, Weghofer A, Wang Q, Zhang L, Albertini DF, Barad DH (2016a). How FSH and AMH reflect probabilities of oocyte numbers in poor prognosis patients with small oocyte yields. Endocrine.

[r10] Gleicher N, Kushnir VA, Sen A, Darmon SK, Weghofer A, Wu YG, Wang Q, Zhang L, Albertini DF, Barad DH (2016b). Definition by FSH, AMH and embryo numbers of good-, intermediate- and poor-prognosis patients suggests previously unknown IVF outcome-determining factor associated with AMH. J Transl Med.

[r11] Gomez R, Schorsch M, Hahn T, Henke A, Hoffmann I, Seufert R, Skala C (2016). The influence of AMH on IVF success. Arch Gynecol Obstet.

[r12] Goswami M, Nikolaou D (2017). Is AMH level, independent of age, a predictor of live birth in IVF?. J Hum Reprod Sci.

[r13] Hazout A, Bouchard P, Seifer DB, Aussage P, Junca AM, Cohen-Bacrie P (2004). Serum antimüllerian hormone/müllerian-inhibiting substance appears to be a more discriminatory marker of assisted reproductive technology outcome than follicle-stimulating hormone, inhibin B, or estradiol. Fertil Steril.

[r14] Irez T, Ocal P, Guralp O, Cetin M, Aydogan B, Sahmay S (2011). Different serum anti-Müllerian hormone concentrations are associated with oocyte quality, embryo development parameters and IVF-ICSI outcomes. Arch Gynecol Obstet.

[r15] Jacobs C, Nicolielo M, Erberelli R, Mendez F, Fanelli M, Cremonesi L, Aiello B, Lorenzon AR (2020). Correlation between morphokinetic parameters and standard morphological assessment: what can we predict from early embryo development A time-lapse-based experiment with 2085 blastocysts?. JBRA Assist Reprod.

[r16] Kotanidis L, Asimakopoulos B, Nikolettos N. (2013). Association between AMH, oocyte number and availability of embryos for cryopreservation in IVF. In Vivo.

[r17] La Marca A, Sighinolfi G, Radi D, Argento C, Baraldi E, Artenisio AC, Stabile G, Volpe A (2010). Anti-Mullerian hormone (AMH) as a predictive marker in assisted reproductive technology (ART). Hum Reprod Update.

[r18] Lamazou F, Genro V, Fuchs F, Grynberg M, Gallot V, Achour-Frydman N, Fanchin R, Frydman R (2011). Serum AMH level is not a predictive value for IVF in modified natural cycle: analysis of 342 cycles. J Gynecol Obstet Biol Reprod..

[r19] Lehmann P, Vélez MP, Saumet J, Lapensée L, Jamal W, Bissonnette F, Phillips S, Kadoch IJ (2014). Anti-Müllerian hormone (AMH): a reliable biomarker of oocyte quality in IVF. J Assist Reprod Genet.

[r20] Majumder K, Gelbaya TA, Laing I, Nardo LG (2010). The use of anti-Müllerian hormone and antral follicle count to predict the potential of oocytes and embryos. Eur J Obstet Gynecol Reprod Biol.

[r21] Mayo JC, Sainz RM, Tan DX, Hardeland R, Leon J, Rodriguez C, Reiter RJ (2005). Anti-inflammatory actions of melatonin and its metabolites, N1-acetyl-N2-formyl-5-methoxykynuramine (AFMK) and N1-acetyl-5-methoxykynuramine (AMK), in macrophages. J Neuroimmunol..

[r22] Nelson SM (2013). Biomarkers of ovarian response: current and future applications. Fertil Steril.

[r23] Nelson SM, Aijun S, Ling Q, Tengda X, Wei X, Yan D, Yanfang W, Zenghui T, Xinqi C, Fraser A, Clayton GL (2020). Ethnic discordance in serum anti-Müllerian hormone in healthy women: a population study from China and Europe. Reprod Biomed Online.

[r24] Palmer CB, Forstein DA, 3rd Higdon HL, Boone WR (2011). Changes in long luteal protocol affects the number of days of stimulation: evolution of an assisted reproductive technology practice. J Reprod Med.

[r25] Pankhurst MW (2017). A putative role for anti-Müllerian hormone (AMH) in optimising ovarian reserve expenditure. J Endocrinol.

[r26] Scheffer JAB, Scheffer B, Scheffer R, Florencio F, Grynberg M, Lozano DM (2018). Are age and anti-Müllerian hormone good predictors of ovarian reserve and response in women undergoing IVF?. JBRA Assist Reprod.

[r27] Smeenk JM, Sweep FC, Zielhuis GA, Kremer JA, Thomas CM, Braat DD (2007). Antimüllerian hormone predicts ovarian responsiveness, but not embryo quality or pregnancy, after in vitro fertilization or intracyoplasmic sperm injection. Fertil Steril.

[r28] Sun TC, Zhang Y, Li HT, Liu XM, Yi DX, Tian L, Liu YX (2018). Sperm DNA fragmentation index, as measured by sperm chromatin dispersion, might not predict assisted reproductive outcome. Taiwan J Obstet Gynecol.

[r29] Sunkara SK, Khalaf Y, Maheshwari A, Seed P, Coomarasamy A (2014). Association between response to ovarian stimulation and miscarriage following IVF: an analysis of 124 351 IVF pregnancies. Hum Reprod.

[r30] Tehraninezhad ES, Mehrabi F, Taati R, Kalantar V, Aziminekoo E, Tarafdari A (2016). Analysis of ovarian reserve markers (AMH, FSH, AFC) in different age strata in IVF/ICSI patients. Int J Reprod Biomed.

[r31] Vural B, Cakiroglu Y, Vural F, Filiz S (2014). Hormonal and functional biomarkers in ovarian response. Arch Gynecol Obstet.

